# Long-term efficacy and safety of Canakinumab in active Hyper-IgD syndrome (HIDS): results from an open-label study

**DOI:** 10.1186/1546-0096-13-S1-O58

**Published:** 2015-09-28

**Authors:** JI Aróstegui, J Anton, I Calvo, A Robles, A Speziale, Y Joubert, G Junge, J Yagüe

**Affiliations:** 1 Hospital Clinic, Barcelona, Spain; 2 Hospital Sant Joan de Déu, Barcelona, Spain; 3 Hospital La Fe, Valencia, Spain; 4 Hospital La Paz, Madrid, Spain; 5 Novartis Pharma AG, Basel, Switzerland; 6 Hospital Clinic, Spain, Spain

## Introduction

Hyper-IgD with periodic fever syndrome (HIDS) is an autoinflammatory disease characterized by periodic episodes of fever, abdominal distress, joint pain, and skin rashes. IL-1 blockade was previously reported as effective in reducing the frequency of episodes and improving clinical symptoms.[^1^,^2^] We report the results of the study assessing the efficacy and safety of canakinumab, an anti-IL-1β human monoclonal antibody, in patients with active HIDS.

## Objectives

The primary objective was to assess the reduction of frequency of flares during the 6-month treatment period compared to a historical period (HP). Secondarily, assessments of reduction in frequency of flares during 24-month long-term follow-up and adverse events (AE) were conducted.

## Patients and methods

This was an open-label, single treatment arm study to assess the efficacy and safety of canakinumab in HIDS patients aged ≥2 years with biallelic *MVK* mutations. The study included a 6-month treatment period (6TP) with up to 6-month withdrawal period (WP) and 24-month long-term treatment period (24TP).

## Results

All enrolled patients (n=9) completed the 6TP and the WP, with 8 completing the 24TP. The median number of flares decreased from 5 (3-12) during HP to 0 (0-2) during 6TP. The median remained at 0 (0-3) until the end of study. During the 24TP, the median flare duration was 3.5 days (2-8, first year) and 8.5 days (6-11, second year). Flare severity remained ‘mild’ to ‘moderate’ at baseline and decreased to ‘mild’ or ‘minimal’ signs/symptoms” and to ‘mild’ or without signs/symptoms” at the first and second year of the 24TP, respectively. Physician's global assessment scores for HIDS disease control changed from either ‘no control’ or ‘poor control’ at baseline to ‘good’ or ‘excellent control’ by Day 4 and were maintained until the end of study. CRP and SAA plasma levels normalized by Day 15 and remained normal thereafter. The most frequent AEs were infections, with four patients experienced 14 mild to moderate SAEs. No AEs were drug-related nor led to discontinuation of study treatment.

## Conclusions

Canakinumab markedly reduced the frequency of flares, rapidly alleviated signs and symptoms of acute episodes and normalized the serological inflammatory markers. The safety profile is consistent with other canakinumab studies. These data support a safe and maintained disease control and reinforce the ongoing development of canakinumab in this therapeutic area.
